# Masked Primary Hyperparathyroidism by Empagliflozin Use

**DOI:** 10.7759/cureus.24488

**Published:** 2022-04-26

**Authors:** Mariam Awada, Zeinab Melhem, Zeinab M Khalaf, Yusef Hazimeh

**Affiliations:** 1 Endocrinology and Diabetes, Faculty of Medical Sciences, Lebanese University, Beirut, LBN; 2 Endocrinology, Diabetes, and Metabolism, Faculty of Medical Sciences, Lebanese University, Beirut, LBN; 3 Endocrinology, Faculty of Medical Sciences, Lebanese University, Beirut, LBN

**Keywords:** masked primary hyperparathyroidism, hyperparathyroidism, hypercalcemia, antidiabetic drugs, empagliflozin

## Abstract

Sodium-glucose cotransporter-2 inhibitors are drugs that regulate blood sugar by decreasing glucose reabsorption from the proximal renal tubules. Primary hyperparathyroidism masked by empagliflozin is very rare and only a few cases are reported in the literature. We report a case of a 57-year-old man with a known history of diabetes on empagliflozin for two years who presented with hypercalcemia and equivocal parathyroid hormone level. Upon cessation of this medication, he had persistent hypercalcemia with a raised parathyroid level, which confirmed the diagnosis of primary hyperparathyroidism. We believe this case is one of the first cases reported in the literature.

## Introduction

Empagliflozin is a sodium-glucose cotransporter-2 (SGLT2) inhibitor that has been approved for the treatment of type 2 diabetes mellitus. It acts by reducing renal reabsorption of glucose and increasing its urinary excretion. This effect at the kidney level increases the level of glucose in the urine, which carries the risk of facilitating urinary tract infections and mycotic infections [1]. Euglycemic diabetic ketoacidosis is common with SGLT2 inhibitors due to decreased plasma glucose levels and decreased insulin release [1].

Hypercalcemia associated with SGLT2 inhibitor use remains rarely reported in the literature. To our knowledge, only three papers reviewed this subject in the available literature written in the English language. All of them are case reports and case series, with a total of five patients [2]. We present herein a case of primary hyperparathyroidism masked by the use of empagliflozin.

## Case presentation

A 57-year-old man with a history of type 2 diabetes mellitus, hypertension, coronary artery disease, and dyslipidemia presented for routine follow-up and was found to have hypercalcemia with a calcium level of 10.9 mg/dL corrected for albumin (normal: 8.8-10.6 mg/dL), repeated twice. The patient denied any calcium supplement consumption other than smoking. Two years prior, he was started on empagliflozin and metformin therapy for his diabetes. Other home medications include aspirin, clopidogrel, bisoprolol, ramipril, Lipitor, and vitamin D. He had a family history of osteoporosis. Vital signs and physical examination were within normal limits. The detailed workup is shown in Table 1.

**Table 1:** Patient's workup

Test	Result
Parathyroid hormone level (pg/mL)	First measurement: 21 (normal: 9-39 pg/mL); repeated in another lab: 13.8 (normal: 15-76 pg/mL)
Calcium level (mg/dL)	First measurement: 10.9 mg/dL (normal: 8.8-10.6 mg/dL); repeated in another lab: 10.9 mg/dL (normal: 8.8-10.6 mg/dL)
25-OH vitamin D level (ng/ml)	23.8 (normal: 30-100 ng/ml)
Phosphorus level (mg/dl)	2.8 (normal: 2.8-4.5 mg/dl)
Thyroid-stimulating hormone level (mU/L)	1.6 (normal: 0.4-4,5 mU/L)
Angiotensin-converting enzyme level (U/L)	30 (normal: 12-68 u/L)
Serum immunofixation	Normal
Urine immunofixation	Normal
Dual-energy X-ray absorptiometry (DEXA) scan	Normal bone density, T score - 0.5, hip - 0.7, L3L4 spine

Empagliflozin was stopped for four weeks, and the repeated calcium level was still high (10.9 mg/dl), but the parathyroid hormone (PTH) level rose to 50.2 pg/ml and 52 pg/ml on two consecutive days. The 24-hour urinary calcium excretion level was 415 mg/24 hours (normal: 0-300). So parathyroid sestamibi scan was performed and showed a focal area of persistently increased uptake on the sestamibi images posterior to the mid aspect of the left hemithyroid consistent with a parathyroid adenoma. The patient underwent lower right parathyroidectomy. Intraoperative PTH dropped from 52 pg/ml to 17 pg/ml and calcium level dropped from 10.9 to 10.2 mg/dl. Histopathology confirmed the diagnosis of parathyroid adenoma.

## Discussion

The primary mechanism of action of SGLT2 inhibitors is at the level of the kidney, where they block the sodium/glucose cotransporters, responsible for the reabsorption of 90% of glucose. This will decrease the glycemia/glucosuria ratio. As a result, a high electrochemical gradient is generated, which will facilitate the reabsorption of phosphate. This results in an increase in the serum level of phosphate, which in turn triggers fibroblast growth factor-23 (FGF-23) secretion [3], a molecule that is known to decrease the level of 1,25-dihydroxyvitamin D [4]. The decreased inhibitory biofeedback caused by a low level of 1,25-dihydroxyvitamin D leads to high PTH secretion [4]. On the other hand, SGLT2 inhibitors cause an indirect increase in PTH and FGF-23 levels when it generates an increased urinary calcium excretion [4].

When compared to placebo, dapagliflozin caused a 16% increase in PTH level, 9% increase in serum phosphate level, and 19% increase in FGF-23 level [5,6]. However, its effect on 1,25-dihydroxyvitamin D and calcium levels was not found to be statistically significant [6]. Blau et al. in their randomized crossover study, supported these results when they studied the canagliflozin effect [7]. In contrast, Filippatos et al. did not found a significant correlation between canagliflozin and PTH levels [8].

The effect of SGLT2 inhibitors on the ionic balance was subject to a meta-analysis conducted by Tang et al. [9]. They found a significant correlation between each type of SGLT2 inhibitor and hypermagnesemia. Dapagliflozin was, however, the only one to significantly increase phosphate levels. Similar to a previous study, this study also concluded no correlation between any type of SGLT2 inhibitors and calcium levels [9].

To our knowledge, only three papers reported hypercalcemia after SGLT2 inhibitors in the available literature written in the English language. All of them are case reports and case series, with a total of five patients. The first case was reported by Kaur and Winters (2015) in a 60-year-old male patient who got a PTH independent hypercalcemia after canagliflozin treatment [4]. Vidas et al. described a similar condition in a 63-year-old woman who was treated with dapagliflozin and thiazide. The authors successfully treated her by discontinuing thiazide [9]. While Akhanli et al. reported hypercalcemia and primary hyperparathyroidism developed in a 49-year-old man diagnosed after dapagliflozin treatment and corrected calcium level turned to normal after right parathyroidectomy [9].

We know that SGLT2 inhibitors cause an increase in PTH levels, and in our case, we should expect that, but a low PTH level with an increase in calcium level developed after using empagliflozin, while hypercalcemia did not regress despite interruption of the medicine, and the PTH level rose two times. This case encourages clinicians to consider SGLT2 inhibitors in their differential diagnosis, as a potential causative agent of hypercalcemia when other common etiologies of primary hyperparathyroidism have been excluded.

The reason why the primary hyperparathyroidism became obvious after stopping empagliflozin remains unclear. Empagliflozin may have normalized phosphorus levels in our patient through its mechanism of phosphorus retention. In addition, volume depletion caused by SGLT2 inhibitors may also contribute to hypercalcemia.

This is the first case described of masked primary hyperparathyroidism associated with the use of empagliflozin.

## Conclusions

SGLT2 inhibitors are a category of antidiabetic drugs that are increasingly being used for type 2 diabetes. Their use is not without side effects. Our case tries to increase awareness about one of these side effects, which is their ability to mask primary hyperparathyroidism. In patients with similar side effects, changing the of type antidiabetic drug may be the appropriate next step to do.
